# Supplementary material for: Resolving the full spectrum of human genome variation using Linked-Reads
Source: Genome Res. 2019 Apr;29(4):635–45. doi: 10.1101/gr.234443.118 (PMC6442396; doi:10.1101/gr.234443.118)
Supplement: Supplemental Material [file supp_gr.234443.118_Supplemental_longranger-2.2.zip › longranger-2.2.tar.bz2!/lr2.2-public/lib/stan/cmdstan/stan/doc/api/html/classstan_1_1common_1_1recorder_1_1values.html]

Stan: stan::common::recorder::values< InternalVector > Class Template Reference


|  |  |
| --- | --- |
|  | Stan  2.5.0  probability, sampling & optimization |


- Main Page
- Related Pages
- Namespaces
- Classes
- Files

- Class List
- Class Index
- Class Hierarchy
- Class Members

All Classes Namespaces Files Functions Variables Typedefs Enumerations Enumerator Friends Macros Pages

- stan
- common
- recorder
- values

Public Member Functions |
List of all members

stan::common::recorder::values< InternalVector > Class Template Reference

`#include <values.hpp>`

|  |  |
| --- | --- |
| Public Member Functions | |
|  | values (const size\_t N, const size\_t M) |
|  | |
|  | values (const std::vector< InternalVector > &x) |
|  | |
| void | operator() (const std::vector< std::string > &x) |
|  | |
| template<class T > | |
| void | operator() (const std::vector< T > &x) |
|  | |
| void | operator() (const std::string x) |
|  | |
| void | operator() () |
|  | |
| bool | is\_recording () const |
|  | |
| const std::vector  < InternalVector > & | x () const |
|  | |

## Detailed Description

### template<class InternalVector> class stan::common::recorder::values< InternalVector >

Definition at line 14 of file values.hpp.

## Constructor & Destructor Documentation

template<class InternalVector >

|  |  |  |  |
| --- | --- | --- | --- |
| stan::common::recorder::values< InternalVector >::values | ( | const size\_t | *N*, |
|  |  | const size\_t | *M* |
|  | ) |  |  |

inline

Definition at line 23 of file values.hpp.

template<class InternalVector >

|  |  |  |  |  |  |
| --- | --- | --- | --- | --- | --- |
| stan::common::recorder::values< InternalVector >::values | ( | const std::vector< InternalVector > & | *x* | ) |  |

inline

Definition at line 31 of file values.hpp.

## Member Function Documentation

template<class InternalVector >

|  |  |  |  |  |
| --- | --- | --- | --- | --- |
| bool stan::common::recorder::values< InternalVector >::is\_recording | ( |  | ) | const |

inline

Definition at line 55 of file values.hpp.

template<class InternalVector >

|  |  |  |  |  |  |
| --- | --- | --- | --- | --- | --- |
| void stan::common::recorder::values< InternalVector >::operator() | ( | const std::vector< std::string > & | *x* | ) |  |

inline

Definition at line 38 of file values.hpp.

template<class InternalVector >

template<class T >

|  |  |  |  |  |  |
| --- | --- | --- | --- | --- | --- |
| void stan::common::recorder::values< InternalVector >::operator() | ( | const std::vector< T > & | *x* | ) |  |

inline

Definition at line 41 of file values.hpp.

template<class InternalVector >

|  |  |  |  |  |  |
| --- | --- | --- | --- | --- | --- |
| void stan::common::recorder::values< InternalVector >::operator() | ( | const std::string | *x* | ) |  |

inline

Definition at line 51 of file values.hpp.

template<class InternalVector >

|  |  |  |  |  |
| --- | --- | --- | --- | --- |
| void stan::common::recorder::values< InternalVector >::operator() | ( |  | ) |  |

inline

Definition at line 53 of file values.hpp.

template<class InternalVector >

|  |  |  |  |  |
| --- | --- | --- | --- | --- |
| const std::vector<InternalVector>& stan::common::recorder::values< InternalVector >::x | ( |  | ) | const |

inline

Definition at line 61 of file values.hpp.

---

The documentation for this class was generated from the following file:

- src/stan/common/recorder/values.hpp


---

[ Stan Home Page ]

*© 2011–2014,
Stan Development Team.*
